# Neoantigen-specific TCR-T cell-based immunotherapy for acute myeloid leukemia

**DOI:** 10.1186/s40164-022-00353-3

**Published:** 2022-11-16

**Authors:** Weijun Zhou, Jinyi Yu, Yilu Li, Kankan Wang

**Affiliations:** 1 Department of Hematology, Zhujiang Hospital, Southern Medical University, Guangzhou, 510282 China; 2 Shanghai Institute of Hematology, State Key Laboratory of Medical Genomics, National Research Center for Translational Medicine at Shanghai, Ruijin Hospital Affiliated to Shanghai Jiao Tong University School of Medicine, Shanghai, 200025 China

## Abstract

Neoantigens derived from non-synonymous somatic mutations are restricted to malignant cells and are thus considered ideal targets for T cell receptor (TCR)-based immunotherapy. Adoptive transfer of T cells bearing neoantigen-specific TCRs exhibits the ability to preferentially target tumor cells while remaining harmless to normal cells. High-avidity TCRs specific for neoantigens expressed on AML cells have been identified in vitro and verified using xenograft mouse models. Preclinical studies of these neoantigen-specific TCR-T cells are underway and offer great promise as safe and effective therapies. Additionally, TCR-based immunotherapies targeting tumor-associated antigens are used in early-phase clinical trials for the treatment of AML and show encouraging anti-leukemic effects. These clinical experiences support the application of TCR-T cells that are specifically designed to recognize neoantigens. In this review, we will provide a detailed profile of verified neoantigens in AML, describe the strategies to identify neoantigen-specific TCRs, and discuss the potential of neoantigen-specific T-cell-based immunotherapy in AML.

## Introduction

Acute myeloid leukemia (AML) is the most common indication for hematopoietic stem cell transplantation (HSCT) [1, 2]. The curative potential of the T cell-mediated graft-versus-leukemia (GVL) effects after HSCT indicates that T cell-based immunotherapy may be a powerful strategy against AML cells [3]. The adoptive transfer of T cells, notably chimeric antigen receptor (CAR)- and T cell receptor (TCR)-engineered T cells, has demonstrated an amazing anti-leukemic potential in mice models and human research [4–8]. TCR-T cells kill leukemic cells through the interaction between their modified TCRs and antigens presented by human leukocyte antigen (HLA) molecules on the surfaces of leukemic cells. TCR-T cells targeting tumor-associated antigens (TAAs), such as WT1, PRAME, and HA-1, exert anti-leukemic effects in vitro and in clinical studies with high-risk AML patients post-HSCT [9–12]. However, owing to low antigen expression in non-malignant cells, antitumor immunity targeting TAAs, also known as “self-antigens”, might be accompanied by serious toxicities [13, 14]. Therefore, a major obstacle to TCR-T cell-based immunotherapy is the selection of appropriate targets. Unlike TAAs, neoantigens arise from somatic mutation and are restricted to malignant cells [15]. Immune escape by neoantigens is less plausible since tumor cells need to express these driver genes to retain their malignant phenotypes [16]. Neoantigens can be recognized as foreign antigens by autologous T cells and thus constitute ideal targets for immunotherapy [17]. T cells specific for neoantigens have been detected in AML patients and in donor T cells after HSCT [18, 19]. Furthermore, specific T-cell activities can also be induced in vitro by epitopes derived from neoantigens using peripheral blood mononuclear cells (PBMCs) from AML patients [20]. These results support the presumption that neoantigen-specific T cell-based immunotherapy represents an effective approach to treating AML patients without harming normal tissues. In this review, we provide an overview of known neoantigens in AML, describe TCR-based immunotherapies targeting certain neoantigens, and discuss the current efforts to translate these findings into the clinic.

## Neoantigens in AML

Recent developments in sequencing technology have revealed that a stepwise accumulation of genetic abnormalities is a hallmark of cancer. Gene fusions, small insertions and deletions (indels), non-synonymous point mutations, and cancer-specific splicing variants can result in the production of neoantigens (Table 1) [16]. One high-quality neoantigen may be sufficient for disease control or perhaps a cure in the majority of instances, if it is derived from the mutation that plays a critical role in the malignant phenotype [21, 22]. AML individuals commonly harbor recurrent gene alterations, which may lead to shared neoantigens that trigger potent anti-leukemic responses [23, 24].

**Table 1:** Potential neoantigens derived from various genetic variations for AML

Gene name	Mutated position	neopeptide	HLA restriction	References
*Gene fusion*				
CBFB-MYH11	/	REEMEVHEL	B^*^40:01	[53]
PML-RARA	/	NSNHVASGAGEAAIETQSSSSEEIV	DR^*^11	[56]
DEK-CAN	/	TMKQICKKEIRRLHQY	DR^*^53; DR^*^9	[101]
*Indels*				
NPM1	283–291	AIQDLCLAV	A^*^02:01	[18, 20]
	283–291	AIQDLCVAV	A^*^02:01	[18, 20]
	289–297	LAVEEVSLR	A^*^02:01	[61]
	290–298	AVEEVSLRK	A^*^02:01	[61]
	288–296	CLAVEEVSL	A^*^02:01	[62]
FLT3-ITD	/	YVDFREYEYY	A^*^01:01	[68]
*Non-synonymous point mutations*				
IDH1	R132H	GWVKPIIIGHHAYGDQYRAT	DRB1^*^0101	[74]
KRAS	G12D	GADGVGKSA	C^*^08:02	[83]
	G12D	GADGVGKSAL	C^*^08:02	[83]
	G12D	VVVGADGVGK	A^*^11:01	[102]
	G12V	VVVGAVGVGK	A^*^11:01	[102]
TP53	R175H	HMTEVVRHC	A^*^02:01	[89, 94]
	R175H	YKQSQHMTEVVRHCPHHERCSDSDG	Class II	[89]
	R175H	VVRHCPHHERCSDSD	DRB1^*^13:01	[89]
	R175H	QHMTEVVRHCPHHER	DRB1^*^13:01	[89]
	Y220C	RNTFRHSVVVPCE	DRB1^*^04:01	[89]
	Y220C	VVPCEPPEV	A^*^02:01	[89]
	Y220C	NTFRHSVVVPCEPPE	DRB3^*^02:02	[89]
	G245S	HYNYMCNSSCMGSMN	DRB3^*^02:02	[89]
	R248Q	YMCNSSCMGGMNQRPILTIITLEDS	Class I and II	[89]
	R248W	SSCMGGMNWR	A^*^68:01	[89]
	R248W	SCMGGMNWRPILTII	DRB1^*^02:01	[89]
	R248W	FEVRVCACPGRDWRTEEENLRKKGE	Class II	[89]
	G245S	HYNYMCNSSCMGSMN	DRB3^*^02:02:01	[90]
	Y220C	NTFRHSVVVPCEPPE	DRB3^*^02:02:01	[90]
	P190L	ALPQHLIRV	A^*^0201	[91]
	R267P	LLGPNSFEV	A^*^0201	[91]
	A161T	RVRAMTIYKQ	A^*^1101	[93]
*Splicing variants*				
SF3B1	R625C/H/L/S	LLIRWQHFL	A^*^0201	[99]
		AALPILFQV	A^*^0201	[99]
		ALLLQLFTL	A^*^0201	[99]
		ALLPGLPAA	A^*^0201	[99]
		RLPGVLPRA	A^*^0201	[99]

In general, the identification of neoantigens comprises the following procedures. The first step is usually to compile a list of genomic alterations (including somatic mutations, indels, and gene fusions) from next-generation sequencing data, including whole-genome sequencing, whole-exon sequencing, RNA-sequencing, and DNA panel data, and convert them into “neopeptides” with appropriate lengths. The second step is to predict the binding affinity between neopeptides and patient-specific HLA alleles. Many tools are available for this prediction, such as BIMAS [25] for HLA class I prediction, TEPITOPE [26], PickPocket [27], MULTIPRED2 [28], MultiRTA [29] for HLA class II prediction, and RANKPEP [30], SYFPEITHI [31], NetMHCpan [32–35], and IEDB [36] for both HLA class I and II prediction. The third step is the evaluation of the immunogenicity of the predicted neopeptides, which usually includes testing the ability of neoepitope to induce specific T cells, and validate the functions of specific T cells against malignant cells through cytokine-releasing methods and cytotoxic assays. In addition, the rapid progress in high-throughput mass-spectrometry approaches allows for direct identification of HLA-binding neopeptides [37]. Furthermore, to better apply neoantigen-based therapies for the treatment of cancer patients, potential immunogenic neopeptides should be rapidly and accurately screened. Numerous pipelines, including pTuneos [38], ScanNeo [39], INTEGRATE-Neo [40], CloudNeo [41], TIminer [42], TSNAD [43, 44], MuPeXI [45], pVAC-Seq [46], Neopepsee [47], and many others, have been established to implement mutation calling of multifarious sequencing data and affinity prediction of MHC molecular. Various neoantigens have been confirmed through the flexible use of the aforementioned methodologies.

Here we summarize hotspot neoantigens in AML.

### Gene fusions

Gene fusions caused by chromosomal translocations play a significant role in AML, and they are implicated in approximately half of AML patients. Most gene fusions contribute to oncogenicity by altering hematopoietic-specific transcription factors, thus blocking myeloid differentiation at a specific stage. Due to the recurrent nature of fusion genes in AML, neoepitopes derived from these fusion genes may serve as efficient neoantigens for immunotherapy in AML patients.

#### CBFB-MHY11

The core binding factor (CBF) protein complex is the main target of translocations in AML [48]. The inv(16)(p13q22) and the less frequent t(16;16)(p13q22) result in the formation of a CBFB-MYH11 fusion gene that encodes the fusion protein CBFβ-SMMHC [49]. The resultant fusion protein disrupts CBF functions and leads to a block of myeloid differentiation and ultimately to leukemia [50–52]. A nonameric neopeptide (REEMEVHEL) derived from the CBFβ-SMMHC fusion protein has shown considerable immunogenicity in HLA-B^*^40:01 donors. CD8^+^ T cell clones induced by this neopeptide kill AML cell lines and primary human AML cells in vitro and in an in vivo patient-derived murine xenograft (PDX) model, in a CBFβ-SMMHC-specific and HLA-A^*^40:01-restricted manner [53].

#### PML-RARA

The *PML-RARA* fusion gene results from a chromosomal translocation t(15; 17)(q24.1;q12.1), which fuses the *PML* gene on chromosome 15 to the *RARA* gene on chromosome 17 [54]. The encoded PML-RARA protein serves as a driving event of acute promyelocytic leukemia (APL) [55]. The neoepitope BCR1/25 (NSNHVASGAGEAAIETQSSSSEEIV), encompassing the fusion region of the hybrid molecule PML-RARA, is recognized by CD4^+^ T cells in an HLA-DR11-restricted fashion. A T cell clone has been shown to lyse neoepitope-pulsed autologous cells or autologous cells transduced to express the PML-RARA fusion protein [56]. However, no PML-RARA-specific CD4^+^ T-cell responses were seen in four HLA-DR^*^11^+^ APL patients in a different study. Additionally, a DR11-restricted CD4^+^ T-cell clone obtained from a healthy donor unable to identify APL blasts from HLA-DR^*^11 patients since the APL cells lack the ability to present the peptide BCR1/25 [57]. As a result, PML-RARA is still regarded as a possible rather than a confirmed neoantigen.

### Indels

Indels occurring in protein-coding genes frequently lead to frameshift peptides that are highly distinct from self and have reduced susceptibility to self-tolerance mechanisms. These frameshift peptides might be recognized by T-cells and induce cellular immune responses, thus indels might be an ideal source of tumor-derived neoantigens.

#### NPM1

In AML, nucleophosmin (*NPM1*) is among one of the most frequently mutated genes, being present in one-third of newly diagnosed cases [58]. Mutations in NPM1 are mostly restricted to exon 12 and cause a frameshift in the C-terminus, resulting in the replacement of the last 7 amino acids (WQWRKSL) with different residues [59]. Most NPM1 mutants carry a characteristic 4-bp frameshift insertion, with > 95% of mutations occurring between nucleotides 960 and 961. The substitution of different residues in the C-terminus of NPM1 destroys the nuclear export signal and causes the localization of the mutant protein (NPM1c) to the cytoplasm [59, 60]. Neoepitopes from NPM1c are capable of stimulating both CD4^+^ and CD8^+^ T-cell responses [19, 20]. Furthermore, NPM1c-specific T cells have been detected in a relapsed AML patient after receiving donor lymphocyte infusion [18]. The spontaneous appearance and persistence of NPM1c-specific T cells in AML patients may contribute to the maintenance of long-lasting remission [61]. Recently, a neoepitope (CLAVEEVSL) from NPM1c has been identified from HLA-A^*^02:01^+^ AML patients using tandem liquid chromatography-mass spectrometry (LC–MS/MS) technique, and then CD8^+^ T cells specific to this neoepitope have been isolated from healthy individuals. T cells transduced with the same TCR show the specific recognition and lysis of HLA-A^*^02:01-positive NPM1c AML cells [62]. As a result, the immunogenicity of NPM1c could be harnessed for immunotherapeutic approaches, including vaccine and adoptive T-cell therapy.

#### FLT3-ITD

Internal tandem duplication (ITD) mutations in the FMS-like tyrosine kinase 3 (*FLT3*) gene are present in about 30% of AML patients [63]. FLT3-ITD is a constitutively activated variant of the FLT3 tyrosine kinase receptor that enhances cellular proliferation and reduces apoptosis of hematopoietic blasts, leading to relatively poor clinical outcome and a higher risk of relapse in AML [64–66]. Duplication regions of FLT3-ITD most likely encode immunogenic neoepitopes that can be presented by individual HLA alleles. Neopeptides derived from FLT3-ITD of several AML cell lines or patients have been predicted with different affinity to HLA-A (A1, A2, A11, and A24) or HLA-B27, some of which showed intermediate or high affinity to HLA-A2 in a T2 cell-based assay [67]. Furthermore, CD8^+^ T cells from an FLT3-ITD-positive patient showed autologous anti-leukemic response to a neoepitope (YVDFREYEYY) encoded by the ITD protein region, in an HLA-A^*^01:01-restricted and FLT3-ITD-restricted manner [68]. Besides, due to the diversity of insert position of FLT3 and fragment length of ITD, a cohort of FLT3-ITD-positive patients is necessary to study the specific T-cell activities, including CD8^+^ and CD4^+^ T-cell responses to fully understand the potential of FLT3-ITD as an immunotherapeutic target.

### Non-synonymous point mutations

Non-synonymous point mutations, which make up a significant portion of potential neoantigens for cancer immunotherapy, account for more than 90% of the driver mutations in malignancies, including AML. Some neoantigens generated from these mutations have high affinities to certain HLA alleles and are highly immunogenic when tested by vaccination. The mutated sites of these immunogenic peptides may be exposed to the TCR, leading to TCR rearrangement, or creating new anchor residues that increase binding affinities for HLA molecules.

#### IDH1

Missense mutations in isocitrate dehydrogenase 1 (*IDH1*) occur at specific arginine residues within the catalytic active sites of enzymes [69]. The hotspot mutation affects codon 132, resulting in a single amino acid substitution from arginine to histidine (R132H) [69]. IDH1^R132H^ induces a leukemic DNA-methylation signature in a preclinical mouse model [69, 70], that is, similar to that observed in human IDH1-mutant AML [71]. The prognosis of AML patients harboring mutated IDH1 is generally poor [72], with an increased probability of relapse [73]. In syngeneic MHC-humanized mice, a peptide vaccine targeting IDH1^R132H^ induces specific therapeutic MHC class II-restricted responses that are directed towards IDH1^R132H^-positive tumors [74]. The specific therapeutic T-helper cell responses to a corresponding peptide vaccine have also been observed in astrocytoma patients with IDH1^R132H^ in a phase I clinical trial (NCT02454634) [75]. Although there is no reported evidence of immune responses against mutated IDH1 in AML, therapy targeting IDH1^R132H^ should be equally applicable to IDH1^R132H+^ AML patients if the neoepitope is processed and presented appropriately, because the codon distribution of IDH1 mutations is not specific to the tissue origin of cancer.

#### KRAS

Mutations in the Kirsten rat sarcoma viral oncogene homolog (*KRAS*) oncogene have been considered as a driver of tumor initiation and maintenance [76]. The majority of mutant *KRAS* are recurrent “hot-spot” mutations that often occur at codon 12, 13, or 61, with codon 12 being the most frequent site of mutation [77, 78]. Substitution of the amino acid glycine (G) to aspartic acid (D) at this site, hereafter referred to as KRAS^G12D^, is the most frequent *KRAS* mutant in human cancers. In AML, *Kras*^*G12D*^ serves as a cooperating factor to promote myeloid leukemogenesis [79], and a contributor to clinical resistance to targeted inhibitors [80]. Vaccines that target KRAS^G12D^ have been utilized in clinical trials in patients with solid tumors, cooperates with chemotherapy and surgery or not [81]. Dendritic cells, DNA plasmids, viruses, yeast, or bacteria have also been used as delivery vectors in preclinical or clinical trials. Recently, a CD8^+^ T-cell reactivity against KRAS^G12D^ has been detected in tumor-infiltrating lymphocytes (TILs) from a patient with metastatic colon cancer, which could recognize and kill tumors in an HLA-C^*^08:02-restricted, KRAS^G12D^-specific manner [82]. Subsequently, in a phase II clinical trial (NCT01174121), objective regression of metastases has been observed in a patient with metastatic colorectal cancer after infusion of HLA-C^*^08:02-restricted TILs composed of four different T-cell clonotypes that specifically targeted KRAS^G12D^ [21]. Furthermore, crystal structures of TCR-HLA-C complexes have revealed that TCRs of these reactive CD8^+^ T-cells can recognize KRAS^G12D^ nonamer with multiple conserved contacts through shared CDR2β and CDR3α, other than the corresponding wild-type peptide [83]. In addition, in a phase I/II clinical trial (NCT03480152), an mRNA vaccine containing KRAS^G12D^ has shown safety and efficacy in patients with metastatic gastrointestinal cancer. This vaccine can elicit KRAS^G12D^-specific T-cell responses, and the specific TCRs have been verified and may be further used in adoptive T-cells therapy (ACT) in the future [84, 85]. As the ACT of T-cells genetically engineered to express tumor-reactive TCRs can elicit regression of widespread cancer in patients with metastatic disease, the TCRs isolated above may extend TCR gene therapy to HLA-C^*^08:02 patients whose tumor express KRAS^G12D^, including AML patients.

#### TP53

*TP53* mutated AML represents a distinct molecular subclass that has a poor response to chemotherapy and shows uniformly short overall survival (median survivals of 4–6 months) [86, 87]. The majority of *TP53* variants are missense mutations evenly distributed throughout the coding sequence, particularly in the DNA-binding domain of exons 4 to 8 [88]. The p53 proteins encoded by mutant *TP53* can be degraded by the proteasome, processed, and presented by HLA molecular to generate neoantigens recognizable by TCRs. TILs from patients with epithelial cancers have been shown to recognize autologous p53 neoantigens. These responses involve multiple *TP53* mutations, including R175H, Y220C, G245S, R248Q, and R248W, and are restricted by a variety of HLA restriction elements [89, 90]. Interestingly, specific responses against mutated *TP53*, such as A161T, R267P, R175H, Y220C, and R248W, can also be induced in PBMCs from cancer patients with those mutations or from healthy donors without the matching expression [91–93]. Additionally, a peptide derived from p53^R175H^, HMTEVVRHC (mutant amino acid underlined), binds to HLA-A^*^02:01 with high affinity. Specific TCRs that recognize this peptide-HLA complex have been verified from a patient with metastatic colorectal cancer. These TCRs mediate the recognition of a variety of cancer cell lines endogenously expressing p53^R175H^, including ovarian cancer, uterine carcinoma, myeloma, and leukemia [94]. A TCR mimic antibody specific to this HLA-A^*^02:01-restricted p53^R175H^ neoantigen has also been developed. This bispecific antibody can successfully activate T cells to lyse cancer cells that present the same neoantigen both in vitro and in mice [95]. Therefore, mutated *TP53* is a promising target for many types of cancers due to its immunogenic and is a common and shared neoantigen.

### Splicing variants

About 20% of AML patients harbor splicing variants, which represent a molecular feature of adverse AML with clinical implications. Additional messenger RNAs (mRNAs) are produced by splice isoforms, which are caused by retained introns, skipped exons, alternate 5′ or 3′ splice sites, or mutually exclusive exons. These mRNAs may be translated into a variety of proteins, which serves as an excellent source of neoantigens.

#### SF3B1

The *splicing factor 3B subunit 1A* (*SF3B1*) gene encodes a core component of the U2 nuclear ribonucleoprotein complex of the spliceosome [96]. Mutations of *SF3B1* are found in about 5.6%–12.5% of de novo AML patients and are more frequent in secondary AML [23, 97, 98]. The common hotspot mutations include the consecutive HEAT domain of the C-terminal region, K700, or the conserved amino acids K666, H662, R625, and E622. Neoepitopes derived from *SF3B1*^*R625*^-related junctions have been found in tumor cells expressing mutated *SF3B1*, other than cells with wild-type SF3B1. Patients with *SF3B1*^*R625*^ mutations contain CD8^+^ T-cells specific for these neoepitopes, most of which display the effector/memory phenotype. These results indicate that *SF3B1* variant-derived neoepitopes have been recognized by the immune system in patients harboring such mutations [99].

In addition to *SF3B1*, an HLA class I immunopeptidome has been established through the identification of peptides eluted from HLA—I molecules by means of LC–MS/MS. The results indicate that the proteasome-generated spliced peptide pool accounts for one-third of the entire HLA class I immunopeptidome in terms of diversity and one-fourth in terms of abundance [100], further support that spliced peptides represent a distinct pool of neoantigens with particular immunological characteristics.

## Neoantigen-specific TCRs targeting AML cells

Experimental efforts to map TCR repertoires for a particular antigen have benefited from recent developments in immunology and high-throughput TCR sequencing techniques. The prerequisite is to accurately select the tumor-reactive and neoantigen-specific T cells. This section will discuss the sample sources and associated markers that are used to identify neoantigen-specific T cells, as well as the neoantigen-specific TCRs targeting AML cells.

### Sources of neoantigen-specific T cells

Neoantigen-reactive T cells are commonly found in patients with solid or hematologic malignancies. Patient samples, including TILs and peripheral circulating lymphocytes, are the main sources of tumor-specific T cells [22, 68, 103–105]. Peripheral blood of an AML patient in complete remission has been found harboring specific T cells targeting an FLT3-ITD derived neoepitope, which secrets significant amounts of IFN-γ and Granzyme B after receiving the stimulation of this neoepitope [68]. Similarly, both CD4^+^ and CD8^+^ T cells against mutated NPM1 have been found in AML patients [20], and the majority of these cells are effector memory and central memory T cells, which may contribute to the maintenance of long-lasting remissions [61]. Additionally, tumor-specific T cells can also be detected in the body fluids of patients, such as urine, ascites, and pleural effusions [106, 107].

Peripheral blood from healthy donors is a second emerging source for neoantigen-specific T cells, which is less invasive in contrast to tumor biopsies and is thus readily available. It has been shown that healthy donors exhibit more frequent and efficient CALR-mutant-specific T-cell responses compared with CALR-mutant patients [108]. Neoepitopes derived from indels and fusion proteins of AML patients, such as mutated NPM1 and CBFB-MYH11, have also been found to be recognized by T cells from healthy donors in several studies [53, 62]. Therefore, induction of neoantigen-specific T cells from healthy donors may provide a means for adoptive cell therapy of hematologic malignancies.

### Related markers to capture neoantigen-specific T cells

Neoantigen-specific T cells can be identified and isolated from bulk lymphocytes of patients or healthy donors using MHC multimers or tetramers carrying the same neoepitopes. However, it is estimated that just 0.002% of the entire lymphocyte population is made up of these T cells [105]. Several markers on T cell surface or T-cell secreted cytokines have been suggested for the further enrichment of neoantigen-reactive T cell clones.

PD-1, also known as CD279, is characterized as an inhibitory receptor on T cells. After in vitro expansion, tumor-reactive TILs have a greater percentage of PD-1-expressing CD8 + T cells, which produce much higher tumor-specific IFN-γ compared with the PD-1-negative counterparts [109]. Similar to this, the expression of PD-1 on peripheral blood lymphocytes has the potential to be used as a biomarker to detect T cells that are responsive to neoantigens [110]. In contrast to PD-1-negative cells, which are the limiting factor in the tumor specificity of cells produced from bulk CD8^+^ TILs, PD-1-positive T cell subsets have been shown to have advantages for adoptive T-cell treatment [111]. In another research, it was discovered that CD8^+^ TILs express PD-1, TIM-3 (a co-inhibitory receptor restricted to IFN-γ-producing T cells), and LAG-3 (an inhibitory receptor), which can be used to identify the autologous tumor-reactive repertoires, including mutant neoantigenic T cells [112].

4-1BB, also known as CD137 and TNFRSF9, is a costimulatory member of the TNF receptor family and has emerged as an important mediator of survival and proliferation [113, 114]. 4-1BB is essential for the survival of activated and memory CD8^+^ T cells. After TCR stimulation, 4-1BB is transiently upregulated from 12 h to up to 5 days [115]. Epitope discovery based on CD137^+^ selection has proven to be an efficient and sensitive in vitro technique for rapidly identifying and isolating antigen-specific CD8^+^ T cells that present at low frequencies and display heterogeneous functional profiles [115]. Through the enrichment of T cells from TILs by sorting for CD137^+^CD8^+^ markers, 27 TCRs that recognize 14 neoantigens produced by autologous tumor cells have been isolated from 6 patients [116]. Memory T cells targeting the mutated KRAS G12D and KRAS G12V have also been identified and isolated with 4-1BB as the T-cell activation marker in another study [117].

CD39, a transmembrane extracellular ATPase, is widely expressed by regulatory T cells, B cells, and some tumor cells. CD8^+^ T cells with high CD39 expression in TILs exhibit an exhausted phenotype with the reduced production of INF-γ, TNF, IL-2, and high expression of coinhibitory receptors [118]. Expression of genes related to cell proliferation and exhaustion which are characteristics of chronically stimulated T cells is enriched in CD39^+^CD8^+^ TILs [119]. A distinct population of CD8^+^ TILs that co-express CD39 and CD103 possesses an exhausted tissue-resident memory state, is capable of recognizing and eradicating autologous tumor cells in an MHC-class I-dependent manner [120]. Most antitumor neoantigen-reactive TILs are found in the differentiated CD39^+^ state [121]. However, in addition to nonresponders receiving adoptive T cell therapy, responsive patients have also been observed to harbor CD39^−^ stem-like neoantigen-specific TILs [121].

Cytokines, such as IFN-γ and IL-2, can be used as markers for screening neoantigen-specific TCRs. A brand-new approach for identifying neoantigen-specific T cells has been developed recently [122]. It involves co-culturing TILs with antigen-presenting cells that have been pulsed with neoantigens and the single-cell RNA sequencing analysis of T cells. Neoantigen-reactive cells are those expressing high levels of IFN-γ and IL-2. Using this method, neoantigen-specific TCR sequences have been successfully identified from two melanoma and one colorectal cancer patient [123].

Taken together, the sources of neoantigen-specific T cells are diverse but limited in frequency. The status of CD8^+^ T cells is quite heterogeneous, both within and between patients [124]. There is currently no specific method or marker to exclusively recognize neoantigen-specific T cells, making the application of the aforementioned methodologies to identify and enrich such T cells challenging. To produce neoantigen-specific TCRs in particular, these methods may be helpful for enriching and expanding neoantigen-reactive T cells before going on to downstream analysis [125].

## Identification of neoantigen-specific TCRs to AML

IN AML, direct identification of neoantigen-specific T cells from patients is hampered by lymphopenia and/or disease-related immunological dysfunction. T cells specific for certain neoantigens may not be present in adequate numbers in PBMCs isolated from AML patients in remission after chemotherapy. PBMCs from healthy donors have therefore been given priority. Using tetramer technology, a significant percentage of PBMCs from six HLA-A^*^02:01^+^ healthy individuals were directly identified as mutated NPM1-specific T cells. Some of these T cell clones have been effectively expanded and displayed specific cytotoxic activity against AML cells that express mutant NPM1. For example, T cells transferred with the modified TCR sequences (TRAV12-2 and TRBV5-1) expressed by the responder clone show the same specific activity against mutant NPM1 [62]. Similarly, high-avidity CD8^+^ CBFB-MYH11 epitope-specific T cell clones have been isolated by the neopeptide-tetramer from HLA-B^*^40:01^+^ healthy donors and subjected to TCR sequencing to identify the paired α and β chains. CD8^+^ T cells transduced with these high-avidity TCRs exhibit antileukemic activity in vitro [53].

**Fig. 1:**
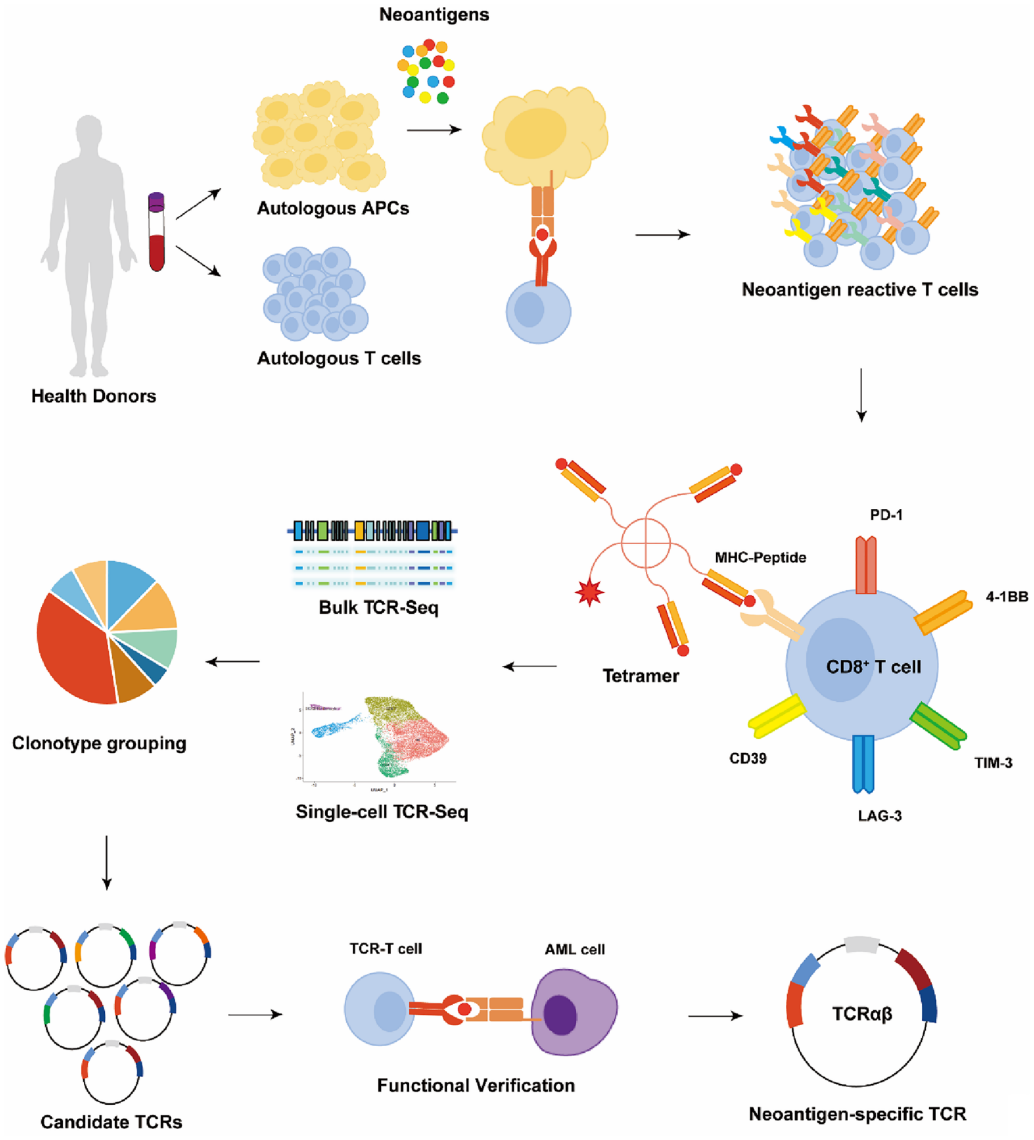
Overview of the procedure for identifying neoantigen-specific TCRs from healthy donors

These findings imply that specific anti-leukemic TCR rearrangements may be induced by neoantigens arising from genetic aberrant events of AML notably indel and fusion and that these changes can be accurately captured by specialized antibodies and TCR sequencing. An important source of neoantigen-specific TCRs for AML, is immune cells from healthy donor since chemotherapy-induced immunological dysfunction in AML patients makes it difficult for them to generate enough anti-leukemic immune responses. Recently, a time-efficient approach to identify pilot neoantigen-specific TCRs from HLA-matched healthy donors has been established, and two of the three chosen TCRs have demonstrated dose-dependent recognition of the corresponding neoantigens [126]. From the time that the neoantigen is stimulated until specific T cells are obtained, this protocol, which could also work for AML, only takes two weeks. Additionally, other than tetramer, immune checkpoints (such as PD-1, TIM3, and 4-1BB), the stem-cell-like marker CD39, and the cytokines mentioned above can also be employed to broaden the screening of neoantigen-specific T-cell for AML (Fig. 1).

## Clinical experience of TCR-based immunotherapy in AML

IT is estimated that about 16% of TCR-T cell-based clinical trials are designed for hematological malignancies, in particular AML (Table 2) [127]. The majority of clinical trials are based on TCR-engineered T cells against WT1, a classic TAA [128]. In patients with relapsed or high-risk leukemia, the administration of WT1-specific donor-derived CD8^+^ cytotoxic T cell clones is well tolerated, and the transferred cells exhibit antileukemic activities in two patients (NCT00052520) [129]. In a subsequent phase 1 clinical trial (NCT01640301), a high-affinity WT1-specific TCR was inserted into Epstein-Bar virus-specific donor CD8^+^ T cells and twelve AML patients with poor-risk prophylactically received these WT1-specific TCR-T cells. The treatments showed significant benefits related to relapse-free survival, overall survival, relapse, and non-relapse mortality [9]. In another first-in-human trial (UMIN000011519), eight patients with refractory AML or high-risk MDS were treated twice with autologous T cells engineered to express a TCR specific for the WT1_234-243_ peptide in an HLA-A^*^24:02-restricted manner, along with siRNAs to eliminate expression of endogenous TCR chains, followed by sequential WT1 peptide vaccines. The TCR-T cells persisted throughout the study period in five patients, four of whom survived more than 12 months [10, 130, 131]. An optimized TCR construct has been established recently, which contains a high-affinity HA-1 specific TCR, a CD8^+^ coreceptor, a safety switch (iCasp9), and a tracking marker. T cells that express this TCR construct are highly functional against HA-1^+^ leukemia cells in vitro, and they were rapidly and completely eliminated using a safety switch [132]. These engineered T cells are being evaluated on patients with hematological malignancies in an ongoing clinical trial (NCT03326921). In addition, clinical trials of TCR-T cell-based therapy that targets PRAME, a well-known cancer-testis antigen, have started in recent years. Two of these trials are NCT022743611 (BPX-701, Bellicum Pharmaceuticals) and NCT03503968 (MDG1011, Medigene AG).

**Table 2:** Clinical trials on engineered TCR-T cells against AML

NCT Number	Target	HLA Allele	Conditioning regimen	TCR Transduced Method	Phase	Participants	Status/results
NCT01640301	WT1	HLA-A2	Cyclophosphamide; Fludarabine	Lentiviral vector	I/II	12	Terminated / RFS* 100% at a median of 44 months after infusion
NCT05066165	WT1	HLA-A^*^02:01	Cyclophosphamide; Fludarabine	CRISPR/Cas9	I/II	54	Active, not recruiting
NCT01621724	WT1	HLA-A^*^02:01	N.A	Retroviral vector	I/II	7	Completed
NCT03326921	HA1	HLA-A^*^02:01	Fludarabine	N.A	I	24	Recruiting
NCT02550535	WT1	HLA-A^*^02:01	Fludarabine; Methylprednisolone	N.A	I/II	3	Completed
NCT02770820	WT1	HLA-A^*^02:01	N.A	N.A	I/II	9	Terminated
NCT02743611	PRAME	HLA-A2	N.A	N.A	I/II	28	Unknown
NCT03503968	PRAME	HLA-A^*^02:01	N.A	N.A	I/II	92	Recruiting

^*^RFS: Relapse-free survival

**Fig. 2:**
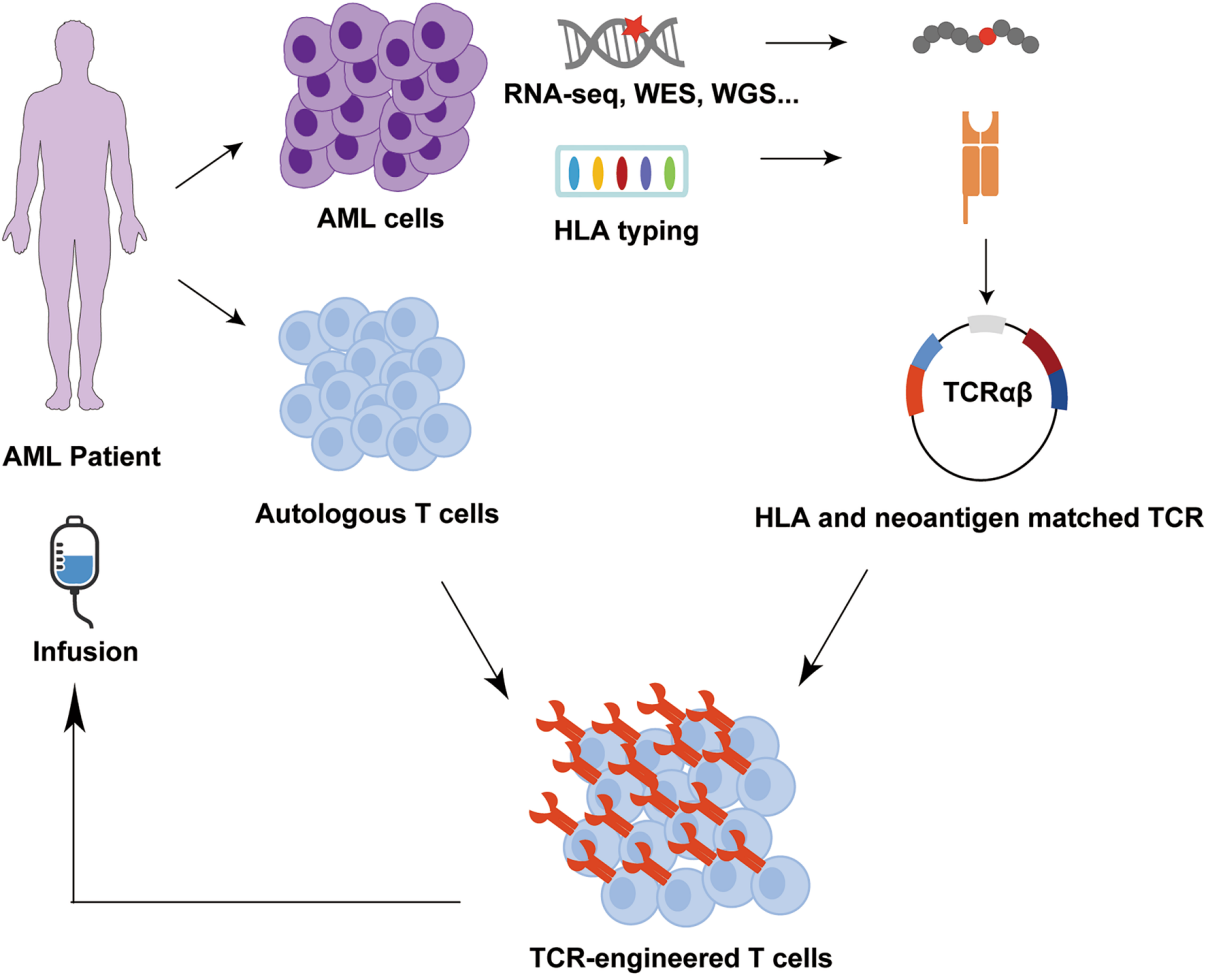
Overview of the neoantigen-specific TCR-based ACT process

Despite the lack of clinical studies for neoantigen-specific TCR-T cell-based therapy in AML, deep and durable tumor shrinkage has been observed in a heavily pretreated patient with metastatic pancreatic ductal adenocarcinoma who received an infusion of autologous T cells transduced with a TCR specific for mutant KRAS^G12D^ [85]. This finding strengthens the clinical application of neoantigen-specific TCR-T cells. Likewise, as more and more TCRs specific to neoantigens that arise from genetic aberrations of AML with different HLA-restriction are being identified in the future, patients who have this particular HLA allele and express the same neoantigens may potentially benefit from this TCR transduction therapy (Fig. 2).

## Comparison of TCR- and CAR-T cell therapy for AML

Another engineered T-cell-based therapy known as CAR-T is frequently employed in clinical trials to treat relapsed or refractory AML patients [133]. Both TCR- and CAR- therapies modify and expand T lymphocytes from patients or donors ex vivo before re-infusing them into the patient’s body to eradicate cancerous cells. Typically, viruses (such as lentiviral and retroviral) or CRISPR/Cas9 are used to introduce TCR or CAR structures into T cells. Both therapies have certain advantages and disadvantages, which have been well-reviewed elsewhere [134, 135]. Other than these, TCR- and CAR-modified T cells differ greatly, as described below.

### Construct

The TCR complex is a heterodimer consisting of alpha and beta chains. Unlike TCR, CARs are synthetic receptors created from a combination of an antibody-derived single-chain variable fragment (scFV), a T-cell receptor-derived CD3ζ domain, a transmembrane domain, and one or more intracellular co-stimulatory domains (such as CD28, 4-1BB/CD137, ICOS, and/or OX40) that have evolved across five generations [136]. Other than that, safety switches have been introduced to TCR and CAR constructs [132, 137]. Modified T cells carrying this construct have been used in clinical trials for the treatment of AML patients to improve safety [132].

### Targets and MHC involvement

The scFV domain of CAR-T cells binds to antigens expressed on the surface of cancer cells in an MHC-independent manner, therefore, targets for CAR-T therapy are all surface proteins, such as CD33 [138], CD123 [139, 140], NKG2D ligand [141], CD70 [142], CD13-TIM3 [4] for AML patients. In contrast to CAR-T, the heterodimer expressed in TCR-T cells recognizes peptides presented by MHC molecules. As a result, the spectrum of targets for TCR-T cells has expanded to include both intracellular and surface antigens. Currently, TCR-T cell therapy for AML patients concentrates on neoantigens, such as NPM1 and CBFB-MYH11, as well as TAAs like WT1, PRAME, and HA-1.

### Toxicities

Modified T cells are potential for “On-target, off-tumor” toxicity in cases where the antigenic target is co-expressed on normal cells, such as CAR-T cells targeting leukemia-associated antigens and TCR-T cells targeting TAAs [136]. Neoantigens are only found in malignant cells, therefore employing them as targets might help with this problem. Other than this, CAR-related toxicities also include cytokine release syndrome and neurological toxicities, which are also referred to as “on/off tissue toxicity” effects and have varying degrees of severity but a similar clinical presentation [143].

## Challenges for the application of TCR-T cells

Although neoantigen-specific T cell-based immunotherapy has shown promising potential in treating AML patients without harming normal tissues, there are still challenges ahead for the application of TCR-T cells. First, the accurate identification of neoantigens presented by HLA molecules on the cell surface is a major obstacle. LC coupled to MS/MS has proved to be a fast and less biased technology to advance the identification of endogenously processed and presented HLA-ligands, although sample handling methods, instrumentations, and data searching algorithms are still required to be improved. Currently, integrating MS HLA-ligand profiling with other “-omic” technologies, such as whole exon sequencing and RNA-sequencing, is indeed of help to identify tumor-specific antigens for AML immunotherapy [37]. Second, the induction of neoantigen-specific TCRs directly from AML patients may be restricted by illness-related immunodeficiency [53, 62]. PBMCs from healthy donors may provide a source to induce neoantigen-specific T cells. Third, the mismatch between exogenous and endogenous chains may hinder the correct expression of modified TCRs on the surface of T cells. Substitution the constant region of the TCR construct with a mouse version may reduce the frequency of alpha and beta chain mismatches [144]. With these improvements, the TCR-T cell application will become feasible on clinical timescales.

## Conclusion

TCR-T cell-based immunotherapy for hematologic malignancies has made significant strides, especially in the treatment of high-risk AML. The major challenge is identifying appropriate targets. A variety of potential TAAs are being studied in preclinical or clinical settings as targets for TCR-T cell therapy, but special attention must be paid to the adverse effects caused by “off-targets”, because TAAs are also expressed in a small number of normal tissues. Neoantigens seem to be the best candidates for TCR-T targets in this regard since their expression is limited to AML cells. Encouragingly, high-avidity TCRs specific for mutant NPM1 and CBFB-MYH11 have been identified from the repertoires of healthy donors. T cells that have been genetically modified to express these TCRs shown specific killing abilities against AML cell lines as well as blast cells harboring mutated NPM1 and CBFB-MYH11, respectively. Given that the majority of AML patients exhibit these neoantigens, the results of future clinical trials are expected. Further research is needed into TCR-T cells that are directed against additional recurrent somatic mutations in AML, including IDH1/IDH2, FLT3-ITD, DNMT3A, and AML1-ETO.

In conclusion, neoantigen-specific TCR-T cell therapy has good potential to treat AML. Illustrating the interaction between TCRs and neoantigens that are responsible for T cell reactivity, anti-leukemic responses, and clinical effects is the cornerstone. The security and efficacy of genetically modified TCR-T cells are vital as well. The present article reviewed advances in neoantigens for AML and proximal new techniques which can identify neoantigen-specific TCRs and activate a T-cell response. There are several restrictions, nevertheless, that need to be addressed in further study.

## Data Availability

Not applicable.

## Abbreviations

ACT: Adoptive T-cells therapy
AML: Acute myeloid leukemia
APL: Acute promyelocytic leukemia
CAR: Chimeric antigen receptor
CBF: Core binding factor
GVL: Graft-versus leukemia
HLA: Human leukocyte antigen
HSCT: Hematopoietic stem cell transplantation
IDH1: Isocitrate dehydrogenase 1
Indel: Insertion and deletion
ITD: Internal tandem duplication
KRAS: Kirsten rat sarcoma viral oncogene homolog
LC–MS/MS: Liquid chromatography–mass spectrometry
PBMC: Peripheral blood mononuclear cell
PDX: Patient-derived murine xenograft
SF3B1: Splicing factor 3B subunit 1A
TAA: Tumor-associated antigens
TCR: T cell receptor
TIL: Tumor-infiltrating lymphocytes
